# Quality of Life and Morbidity after Endoscopic Endonasal Skull Base Surgeries Using the Sinonasal Outcomes Test (SNOT): A Tertiary Hospital Experience

**DOI:** 10.1155/2021/6659221

**Published:** 2021-05-08

**Authors:** Dhaidan M. Alshammari, Ali Almomen, Mahmoud Taha, Hussain Albahrna, Shuruq Alshammari

**Affiliations:** ^1^Otorhinolaryngology Surgery, Ministry of Health, Riyadh, Saudi Arabia; ^2^Rhinology and Endoscopic Skull Base Surgery at King Fahad Specialist Hospital, Dammam, Saudi Arabia; ^3^Neurosurgery Skull Base Surgery at King Fahad Specialist Hospital, Dammam, Saudi Arabia; ^4^Qatif Central Hospital, Qatif, Saudi Arabia; ^5^Medical Student at Northern Border University, Arar City, Saudi Arabia

## Abstract

**Introduction:**

Endoscopic endonasal skull base surgery (EESBS) has been associated with a minimally invasive and effective approach for pathology of the anterior skull base and associated with less overall morbidity compared with open approaches. However, it is associated with its own potential morbidity related to surgical manipulation or resection of normal and noninflamed intranasal structures to gain adequate access. The assessment of sinonasal QOL (quality of life) postsurgery is therefore a vital aspect in follow-up of these patients.

**Objectives:**

To assess quality of life and morbidity after endoscopic endonasal skull base surgery using the Sinonasal Outcomes Test (SNOT-22). *Methodology*. A single-center retrospective cross-sectional review with a sample of 80–100 patients undergoing endoscopic endonasal transsphenoidal surgery was conducted at the ENT and Neurosurgery departments of King Fahad Specialist Hospital-Dammam (KFSH-D) for a period of 10 years from March 2010 to March 2020. Data were collected through hospital records and database, as well as from patients through phone call interviews. Records were reviewed for diagnosis, demographic features, and 22-item Sinonasal Outcomes Test (SNOT-22) scores noted at three points in time: prior to procedure and after, at 3 months and 6 months.

**Results:**

Within the study cohort comprising 96 patients, the mean age of the participants was 39.5 ± 12.1 years, and diagnostic typing before and after histopathological investigations revealed maximum pituitary adenomas (46.9%) closely followed by CSF-related ailments (41.7%). The changes in the mean and standard deviation of the total SNOT-22 scores postoperatively at the 3^rd^ month (9.5 ± 5.4) and the 6^th^ month (8.8 ± 5.2) were statistically significant (*p* < 0.001) when compared to the preoperative score (10.8 ± 5.1).

**Conclusion:**

Although there was a predicted passivity of symptoms in the post-EESBS period, several significant positive outcomes were seen. The increase in discomfort in the sleep domains postsurgery is an issue to pursue and reason out. The overall SNOT-22 scores noted preoperatively and 3 and 6 months postoperatively showed statistically significant improvements in QOL with no long-term effects.

## 1. Introduction

Endoscopic endonasal skull base surgery (EESBS) has been associated with a minimally invasive and effective approach for pathology of the anterior skull base and associated with less overall morbidity compared with open approaches [1]. Significant surgical advances combined with technological development have established the nasal approach as the gold standard for the resection of pituitary tumors and other sellar lesions [2, 3]. The endoscopic approach has been shown to result in shorter hospital stay, with safer and faster recovery rates as well as having fewer complications when compared with open or microscopic approaches [4].

The endonasal approach is associated with its own potential morbidity related to surgical manipulation or resection of normal and noninflamed intranasal structures to gain adequate access. Because the surgical procedure results in varying degrees of destruction of the nasal structure, some nasal symptoms may occur postoperatively such as crusting, rhinosinusitis, epistaxis, hyposmia, or nasal obstruction. Owing to the complicated and extensive nature of surgery, conceptually, the quality of life (QOL) is especially important in those with skull base lesions.

Thus, the impact of endoscopic skull base surgery outcomes on quality of life (QOL) is an active area of investigation in rhinology and skull base surgery. Previous studies have demonstrated negative effects on quality of life on several outcome measures [1, 3, 5]. While several quality of life (QOL) measures have been used for skull base and nasal surgery patients, the Sinonasal Outcomes Test (SNOT) is the most appropriate tool to evaluate and assess sinonasal morbidity duly because it provides valuable and quantifiable data for measuring sinonasal complications as well as morbidity after skull base surgery. The survey includes sinus-specific domains, psychological domains, and sleep domains that assess general health [6, 7].

This study aims to review outcomes in patients having undergone endoscopic skull base surgery in King Fahad Specialist Hospital-Dammam (KFSH-D), a tertiary referral hospital in the eastern province of Saudi Arabia, with a view to assess patient satisfaction after surgery, follow-up improvement and progression of symptoms, improve protocols so as to decrease morbidity and complications, and overall improve quality of life for the postsurgical patient.

### 1.1. Literature Review

Across the healthcare landscape, the quality of healthcare services continues to be a major priority for patients and professionals alike. From the solo provider to the third party, instituting a program to link payment with service outcomes quality is paramount at nearly every level. Tacit understanding exists that measurement of quality is feasible, adducing references that determine the performance of the individual, entity, or process [8].

The collaborative effects of technological advancements and improved endoscopic techniques over the past 2 decades have made the application of endoscopic endonasal conduit commonplace for a myriad range of skull base pathologies. With the advent of this technique of endoscopic endonasal skull base surgery (EESBS), both patients and surgeons have benefitted, with improved surgical outcomes and intraoperative visualization on the one hand and decreased perioperative complications on the other when compared against the traditional approaches [9–12]. Despite this, EESBS proffers unique sinonasal morbidities that perturb the postprocedure quality of life (QOL) [13]. Researchers are constantly attempting to create comprehensive, validated, skull base QOL instruments, although they are not universally accepted yet [7, 14–17]. More often than not, rhinologic specific questionnaires are alone used to assess the impact of EESBS on QOL of the patient albeit with intrinsic shortcomings [18].

Currently, the 22-question Sinonasal Outcomes Test (SNOT-22) is the most commonly wielded sinus-specific QOL instrument to study the morbidity of outcomes after surgery [6, 10, 19]. The Sinonasal Outcomes Test Questionnaire includes 22 questions spread into 4 domains, namely, nasal, otologic, sleep, and emotional [20]. The nasal domain of the SNOT-22 Questionnaire includes eight entities, six rhinologic symptoms (the need to blow nose, sneezing, runny nose, thick nasal discharge, loss of smell/taste, and nasal blockage), and two postrhinologic symptoms (postnasal discharge and cough). The domain concerning sleep also contains eight questions (difficulty sleeping, waking in the middle of the night, lack of a good night's sleep, waking tired, fatigued during the day, reduced productivity, reduced concentration, restlessness, or irritation). In the otologic domain, stuffed ear, dizziness or vertigo, earache, and facial pain or pressure were the 4 entities of concern, while in the emotional domain, the patient was evaluated for sadness or a feeling of shame or frustration [20, 21].

The SNOT-22 is yet to be validated and has inherent shortcomings in its use; nevertheless, it caters as a reliable indicator of sinus-specific symptomatology in patients [18]. To date, pertinent studies have predominantly been marginal, single-institution, retrospective studies showing mixed results, although in general, they revealed a postoperative resurgence in terms of sinonasal QOL [22–31]. Ultimately, an enhanced comprehension of postoperative QOL after an endoscopic endonasal approach will aid in enriched patient counseling and disease management, besides identifying loci for focus in quality improvement initiatives.

## 2. Methodology

With prior approval from the research committee, a retrospective cross-sectional review with a sample of 80–100 patients undergoing endoscopic endonasal transsphenoidal surgery was conducted. This was a single-center study involving the ENT and Neurosurgery departments at King Fahad Specialist Hospital-Dammam (KFSH-D) for a ten-year period from March 2010 to March 2020.

Data were collected through hospital records and databases, as well as from patients through phone call interviews. Oral informed consent was obtained from the participants. Records were reviewed for diagnosis, demographic features, and 22-item Sinonasal Outcomes Test (SNOT-22) scores noted at three points in time: prior to the procedure and after, at 3 months and 6 months. The SNOT-22 score indicated the total score obtained from all the 22 items entailed in the questionnaire.

Using the mean of the completed items, an incomplete SNOT-22 questionnaire could be imputed for item-level missingness, if at least 50% of the items were marked, as conducted in contemporary research, when missing data were encountered in their study [1, 32, 33]. This is also analogous to studies that utilize other patient-based outcome measures to contemplate quality of life scores [8, 34, 35].

The study included all those patients above 18 years who underwent an elective endonasal endoscopic skull base surgery (EESBS) in KFSH-D, with regular follow-up. Any surgery engaging an endonasal endoscopic approach as a treatment for a lesion grossly entailing the bone of the skull base with or without intracranial involvement was considered as EESBS. Those patients not in regular follow-up or surgery performed on an emergency basis or outside KFSH-D were excluded from the study.

The primary outcomes expected were the changes in SNOT-22 scores compared at 3 distinct time markers: prior to the procedure and at 3 months, and at 6 months postoperatively. All baseline characteristics were summarized by frequency and percentage values in case of categorical variables and by calculating mean and standard deviation (SD) for continuous variables.

The data obtained were preserved in a well-locked cabinet in the clinic with access only by the investigators involved in this research. No vulnerable subjects were engaged, and there was no risk to subjects. The anticipated benefits were to follow their improvement after surgery and increase patient satisfaction.

## 3. Results

A total of 96 patients fulfilling the inclusion criteria and were taken up for the study (Table 1). The mean age of the participants was 39.5 ± 12.1 years, with a near equal gender distribution of 49 males (51%) and 47 females (49%).

Diagnostic typing before histopathological investigations showed that most patients had a pituitary adenoma (46.9%) closely followed by CSF-related ailments (41.7%). Craniopharyngiomas (2.1%), clival mass (5.2%), and meningiomas (4.2%) together constituted less than 15% of the total.

Minimal comorbidities were seen in the patients. Only 3 (3.1%) had a CSF leak, while diabetes, right eye blindness, and synechiae were each seen in 1 patient.

**Figure 1:**
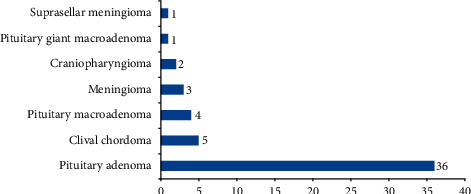
Histopathological final diagnosis.

Histopathological diagnostic typing (Figure 1) was available for only 52 patients. Of them, the most prevalent diagnosis was pituitary adenoma (*n* = 36). Others included clinical chordoma (*n* = 5), pituitary macroadenoma (*n* = 4), meningioma (*n* = 3), craniopharyngioma (*n* = 2), pituitary giant macroadenoma (*n* = 1), and suprasellar meningioma (*n* = 1).

Pre- and postprocedural (3^rd^ and 6^th^ month) SNOT-22 scores were calculated as the number of patient complains for each entity in the four domains (Table 2). Outcomes postsurgery were mostly positive, showing reduced symptoms at both points of time in many categories.

### 3.1. Nasal Domain

In the nasal domain, nasal blockage, runny nose, and decrease/absence of smell showed less numbers (1–12% approximately) at the 3rd month mark and decreased further (1–5% approximately) by the 6^th^ month. Postnasal discharge, however, remained the same even after the surgery, while the need to blow nose and sneezing showed a decrease by just 1% at the 6-month timeline. Sneezing showed an improvement by 5%, but only at the 6-month mark.

### 3.2. Otologic Domain

Patient complaints of dizziness remained the same after surgery (15.5%). The other three (ear fullness, ear pain, and facial pain) were decreased around 2–4% at the 3^rd^ month from prior to surgery, but showed no further change.

### 3.3. Sleep Domain

Here, the outcomes were a mixed bag. Outcomes of difficulty in falling asleep and fatigue showed improvement by ∼2%, but the number of patients waking up at night (13.5%) and lacking a good night sleep (1%) remained constant even after the procedure. A peculiar finding here was that 2 entities (reduced concentration and reduced productivity) showed a negative result, increasing by ∼3% at both the 3^rd^ and 6^th^ month mark after surgery. Similarly, the number of patients complaining of restlessness increased in the 6^th^ month by 3%, although the pre- and 3 month postsurgery outcomes were unchanged (3.1%).

### 3.4. Emotional Domain

In this category, while “frustrated” participants remained the same at all three points of time, the “sad” participants decreased in the 3^rd^ month from 5.2% to 3.1%.

Outcomes of the total SNOT-22 Score at 3^rd^ month and 6^th^ month is given in Table 3.

Within the study cohort comprising 96 patients, the changes in the mean and standard deviation of the total scores postoperatively, at the 3^rd^ month (9.5 ± 5.4), and at the 6^th^ month (8.8 ± 5.2) were statistically significant (*p* < 0.001) when compared to the preoperative score (10.8 ± 5.1).

## 4. Discussion

With the wide view of the endoscope and, thereby, broadened surgical field, the extent of resection and oncologic outcomes have greatly improved, with shorter hospital stays and ease of return to daily activities. However, the nature of the endonasal endoscopic approach solicits some degree of sinonasal morbidity after the procedure, especially in those that transcend a simple transsellar approach [1, 18]. The SNOT-22, a validated and widely used instrument, owing to the parallel nature of endoscopic sinus and endoscopic skull base surgery, could be applied to EESBS with a certain degree of success. Moreover, using a patient-reported instrument to describe the postsurgical QOL gaining popularity as the surgeon's perception has been demonstrated as inaccurate in many a survey [36, 37].

In the present study, the mean age of patients and their gender ratio are akin to similar studies by Choi et al. [1] and Thompson et al. [3]. The majority of lesions identified both before and after histopathological examination were pituitary adenoma (37.5%), which can connate with McCoul et al. [22] and Bhenswala et al. [18], where pituitary adenoma was dominant (57.6% and 56.9%, respectively) with a variety of benign and malignant tumors forming the rest. As endoscopic skull base surgery involves the manipulation of natural corridors of the nasal cavity to arrive at the anterior skull base, the conjecture of practitioners is that this may therefore be more ably tolerated by patients [38, 39]. A measurement of the QOL after EESBS is, ergo, one way to appraise this claim for its merit.

Patients with a preoperative SNOT-22 score <20 at baseline could be considered asymptomatic and were expected to show minimal improvement in postoperative sinonasal QOL as per the SNOT-22 score established norms [40]. Concurring with such expectations, a more robust and sustained melioration in sinonasal QOL was anticipated in patients with poor preoperative scores as opposed to those with few preoperative complaints. In many entities, the near postoperative period (3 months) was characterized by poor sinonasal QOL scores across all domains. This was not necessarily startling given the envisaged post-EESBS nasal crusting, drainage, and congestion [41]. As the SNOT scores were not obtained beyond 6 months in this study, an evaluation to 1 year could not be made.

Utilizing the SNOT-22 instrument, studies by Choi et al. [1] and McCoul et al. [22] demonstrated that in patients who underwent EESBS, no significant changes to QOL scores were experienced at the 3^rd^ month and 6^th^ month after surgery when compared to preoperative scores. This study, in contrast, noted a significant association between these parameters. Amit et al. [42] reported that endoscopic approach was associated with greater QOL scored on the SNOT-22, especially the impact on emotions and physical functions. The objectives in treatment for sinonasal and skull base cancers tether at a balance between achieving maximum oncological control and curtail functional disability of patients to a minimum.

## 5. Limitations

One of the limitations of this study is that the period of follow-up does not extend beyond 6 months and hence cannot chart outcomes for a wider timeline. This study pertains to a single specialist center with a smaller sample, and thereby, it may not be possible to generalize the results obtained. As it was not feasible to follow-up, all the patients underwent the procedure, a bias due to convenience sampling exists that may have interfered with the perceived judgements.

## 6. Conclusion

With the evolution of skull base surgery with endoscopic endonasal approaches, a holistic appraisement of outcomes is vital, with a rigorous measurement of functional outcomes and not limited solely to survival rates. Herein, QOL gains paramount importance. The present study shows that although there is a predicted passivity of symptoms in the post-EESBS period, several significant positive outcomes are seen. The increase in discomfort in the sleep domains after surgery is an issue to pursue and reason out. The overall SNOT-22 scores taken preoperatively and 3 and 6 months postoperatively were found to have improved significantly with no long-term effects.

## 7. Recommendations

Findings from such studies impart a point estimate and/or a range of expected changes after endoscopic surgical procedures, which can invigorate quality improvement initiatives. An attempt to conduct such research with unbiased data and patient level metrics spanning a wide spectrum of both patients and providers, using validated QOL outcome measures, can enhance the understanding of patient outcomes and the morbidities associated with EESBS in conjunction with the accuracy and precision of the score estimate and range. Tumor-specific approaches and the surrounding QOL can also yield quality improvement strategies.

## Figures and Tables

**Table 1:** Demographic characteristics (*n* = 96).

Characteristics	Value
Age in years	
Mean (SD)	39.5 ± 12.1

Gender, *n* (%)	
Male	49 (51.0%)
Female	47 (49.0%)

Type	
Pituitary adenoma	45 (46.9%)
CSF leak	40 (41.7%)
Craniopharyngioma	2 (2.1%)
Clival mass	5 (5.2%)
Meningioma	4 (4.2%)

Complication	
CSF leak	3 (3.1%)
Diabetes	1 (1.0%)
Right eye blindness	1 (1.0%)
Synechiae	1 (1.0%)

**Table 2:** Symptom domains in SNOT-22.

Symptoms	Pre	3^rd^ month	6^th^ month
Nasal domain			
Nasal blockage	42 (43.8)	41 (42.7)	37 (38.5)
Need to blow nose	19 (19.8)	19 (19.8)	18 (18.8)
Runny nose	61 (63.5)	49 (51.0)	47 (49.0)
Postnasal discharge	41 (42.7)	41 (42.7)	41 (42.7)
Sneezing	49 (51.0)	49 (51.0)	45 (46.9)
Cough	17 (17.7)	12 (12.5)	12 (12.5)
Decrease/absence of smell	15 (15.6)	13 (13.5)	10 (10.4)
Thick nasal discharge	23 (24.0)	21 (21.9)	22 (22.9)

Otologic domain			
Ear fullness	10 (10.4)	6 (6.3)	6 (6.3)
Facial pain	11 (11.5)	9 (9.4)	9 (9.4)
Dizziness	15 (15.6)	15 (15.6)	15 (15.6)
Ear pain	9 (9.4)	5 (5.2)	5 (5.2)

Sleep domain			
Wake up at night	13 (13.5)	13 (1.35)	13 (13.5)
Wake up tired	11 (11.5)	8 (8.3)	9 (9.4)
Reduced concentration	8 (8.3)	9 (9.4)	11 (11.5)
Reduced productivity	4 (4.2)	5 (5.2)	7 (7.3)
Lack of a good night sleep	1 (1.0)	1 (1.0)	1 (1.0)
Restless	3 (3.1)	3 (3.1)	6 (6.3)
Difficult falling in asleep	3 (3.1)	1 (1.0)	1 (1.0)
Fatigue	24 (25.0)	22 (22.9)	21 (21.9)

Emotional domain			
Sad	5 (5.2)	3 (3.1)	3 (3.1)
Frustrated	2 (2.1)	2 (2.1)	2 (2.1)

**Table 3:** Total SNOT-22 scores.

Time period	Mean (SD)	*P* value
Pre	10.8 (5.1)	<0.001
3^rd^ month	9.5 (5.4)	
6^th^ month	8.8 (5.2)	

## Data Availability

The data used to support the findings of this study are included within the article.
